# Crush versus Culotte stenting techniques for coronary bifurcation lesions: A systematic review and meta-analysis of clinical trials with long-term follow-up

**DOI:** 10.1097/MD.0000000000014865

**Published:** 2019-04-05

**Authors:** En Chen, Wei Cai, Liang-long Chen

**Affiliations:** a Department of Cardiology, Fujian Medical University Union Hospital; b Provincial Institute of Coronary Artery Disease, Fujian, PR of China.

**Keywords:** coronary bifurcation lesion, Crush, Culotte, meta-analysis, percutaneous coronary intervention

## Abstract

**Background:**

In patients with complex true coronary bifurcation lesions (CBLs), Crush or Culotte stenting has been the commonest approaches of percutaneous coronary intervention (PCI). However, the optimal one remains in debate.

**Methods:**

A systematic review and meta-analysis of cohort studies searched from PubMed, EMBASE, Cochrane Central Register of Controlled Trials (CENTRAL), Chinese National Knowledge Infrastructure (CNKI), VIP information database, and WangFang Data Information Site, to compare the long-term safety and efficacy of PCI with Crush versus Culotte in patients with CBLs. The primary end point was target lesion revascularization (TLR) and secondary end points were a composite of major adverse cardiac events (MACE) including cardiac death (CD), myocardial infarction (MI), stent thrombosis (ST), and target vessel revascularization (TVR) by PCI or bypass surgery, and each individual component at long-term follow-up. Furthermore, omitting each study in turn was used to sensitivity analysis for high heterogeneity of studies.

**Results:**

A total of 7 studies were included to perform a meta-analysis, 3 randomized trials and 4 observational studies with 2211 patients, 1281 treated with Crush and 930 with Culotte. There was no significant difference in TLR and MACE between Crush and Culotte [RR 0.76, 95% CI (0.48–1.23), I^2^ = 57%; RR 0.78, 95% CI (0.47–1.29), I^2^ = 83%, respectively]. ST tended to be lower in patients treated with Crush [RR 0.61, 95% CI (0.37–1.01), I^2^ = 23%]. CD and MI were comparable between the 2 groups [RR 0.80, 95% CI (0.43–1.49), I^2^ = 0%; RR 0.74, 95% CI (0.49–1.13), I^2^ = 32%, respectively]. TVR was also associated with the similar risk [RR 0.76, 95% CI (0.49–1.16), I^2^ = 60%]. However, high heterogeneity was detected for TLR, MACE, and TVR, and the source of heterogeneity was DKCRUSH-III study by Chen, SL.

**Conclusions:**

In the treatment of coronary bifurcation lesions, TLR and MACE were not significant difference between the Crush and Culotte groups, but TLR and MACE were also regarded as high heterogeneity mainly due to better outcomes achieved by DK Crush and there was a trend toward lower ST in the Crush group. Crush, particularly DK Crush, may be superior to conventional Culotte for treatment of CBLs.

**PROSPERO registration number:**

CRD42018111868.

## Abstract 2

Supplemental Digital Content is available in the text

## 1 Introduction

Percutaneous coronary intervention (PCI) of coronary bifurcation lesions (CBLs) is still challengeable for interventionists.^[1]^ Despite that the simple strategies, especially provisional side-branch (SB) stenting, are generally recommended for the majority of CBLs by guidelines or consensus,^[2]^ such simple strategies for true or complex CBLs may not be technically safe and clinically effective due to the potential risk of intraprocedural occlusion of significant branches as well as poor capability of maintaining long-term patency of branches. Therefore, to achieve satisfactory results as treating complex CBLs, complex dual-stenting techniques remain mandatory,^[3,4]^ among which the stenting techniques of Crush and Culotte are the commonest options.

With modification of the conventional techniques of Crush and Culotte, both approaches have been broadly used clinically and many clinical studies have confirmed their own safety and efficacy in treatment of complex CBLs.^[4–10]^ Nevertheless, as a dual-stenting technique, Crush or Culotte is technically complicated and the treatment results may be affected by many factors such as stenting technique per se, operator's experience, device's performance, cardiovascular imaging evaluation, patients’ characteristics, and so on.^[11–13]^ Here raises a question whether Crush and Culotte are equally effective or not. Up to date, there are several studies comparing Crush versus Culotte for treatment of CBLs. However, the optimal one remains in debate. Accordingly, we performed a meta-analysis of cohort studies comparing the long-term clinical outcomes of Crush versus Culotte for treatment of complex CBLs.

## 2 Methods

The study protocol was registered with the PROSPERO international database of prospectively registered systematic reviews in health and social care (CRD42018111868), meanwhile performed following in the PRISMA guidelines.^[14]^

### 2.1 Date sources and search strategy

PubMed, EMBASE, Cochrane Central Register of Controlled Trials (CENTRAL), Chinese National Knowledge Infrastructure (CNKI), VIP information database, and WangFang Data Information Site were searched from the beginning of each database up to October of 2018 by entering “Crush [Title/Abstract] AND Culotte [Title/Abstract].”

### 2.2 Eligibility criteria and study selection

The inclusion criteria of the cohort study were as follows: clinical randomized trials and high quality observational studies comparing Crush versus Culotte stenting techniques for coronary bifurcation lesions. There was no language obstacle for inclusion. Studies were excluded if: comparing stenting techniques without Crush and Culotte stenting techniques; without at least 1 year of follow-up; deficiency of available clinical outcomes of patients treated with Crush and Culotte stenting techniques; not metal stents.

Two investigators (EC, WC) had independently screened titles and abstracts, reviewed full-text articles, and determined the eligibility. In order to retrieve all potential relevant published and unreported materials, we also searched conference proceedings, dissertations, and reference lists. We included randomized trials and observational studies with available clinically follow-up. Disagreements were resolved through consultation with corresponding authors or by consensus.

### 2.3 Data extraction and clinical endpoints

The type of study, year of publication, treatment allocation, age, sex, smoking, diabetes, hypertension, hypercholesterolemia, length and diameter of the implanted stents, use of GP IIb/IIIa inhibitors, final kissing balloon dilatation (FKBD), duration of double antiplatelet therapy (DAPT), the time of follow-up, the Crush or Culotte stenting technique were extracted from the included studies. And reported percentages were recalculated to absolute numbers.

Primary end point was target lesion revascularization (TLR) and secondary end points were major adverse cardiac events (MACE) including cardiac death (CD), myocardial infarction (MI), stent thrombosis (ST), and target vessel revascularization (TVR) by PCI or bypass surgery, and each individual component at long-term follow-up. All end points were defined according to Academic Research Consortium (ARC) definitions.^[15]^

### 2.4 Bias assessment and statistical analysis

Two investigators (EC, WC) had also independently assessed the risk of bias and quality of included studies by using the Cochrane Collaboration Assessment Tool for randomized trials^[16]^ and the Newcastle-Ottawa Scale (NOS) for observational studies. The measure size of the included studies was chosen as risk ratio of treatment effect and publication bias was assessed by Egger test of intercept^[17]^ and Begg test^[18]^ (statistically significant with *P* ≤.1).

Continuous variables were expressed as mean ± SD and discontinuous variables were reported as number (percentage) and relative risk (RR) with 95% confidence interval (95% CI). The heterogeneity was assessed by Cochrane Q chi-square statistics and I^2^ statistics.^[19]^ When I^2^≥50% and Cochrane Q chi^2^ test *P*≤.1, we considered them as lacking of homogeneity and the pooled RR was evaluated by the DerSimonian–Laird method for random effects.^[20]^ When I^2^ < 50%, a fixed effects model was used. Each of endpoints was corresponded to a classic Forest plot, including point estimates and 95% CI.

A sensitivity analysis was performed by omitting each study in turn when heterogeneity was found. The Revman 5.3 free package program and the statistical software package (Stata 14.0) were used for analysis. A 2-tailed *P* value < .05 was considered statistical significance.

## 3 Results

### 3.1 Study characteristics and quality assessment

**Figure 1:**
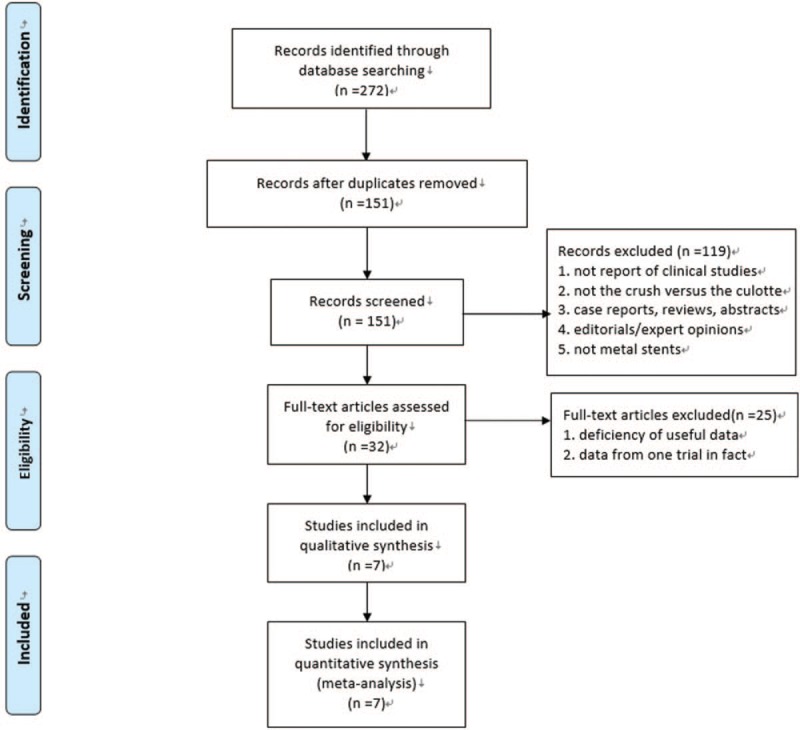
Flow diagram showing the process of study selection.

We had screened and reviewed a total of 272 citations, and finally 7 studies were identified for inclusion and further evaluation. The study process was described in Fig. 1. Six studies^[4–9]^ were published in English, and 1 study^[10]^ in Chinese. Of the included studies, 3 were randomized trials,^[4–6]^ the other 4 were observational studies,^[7–10]^ with a total of 2211 patients, 1281 treated with Crush and 930 with Culotte.

**Figure 2:**
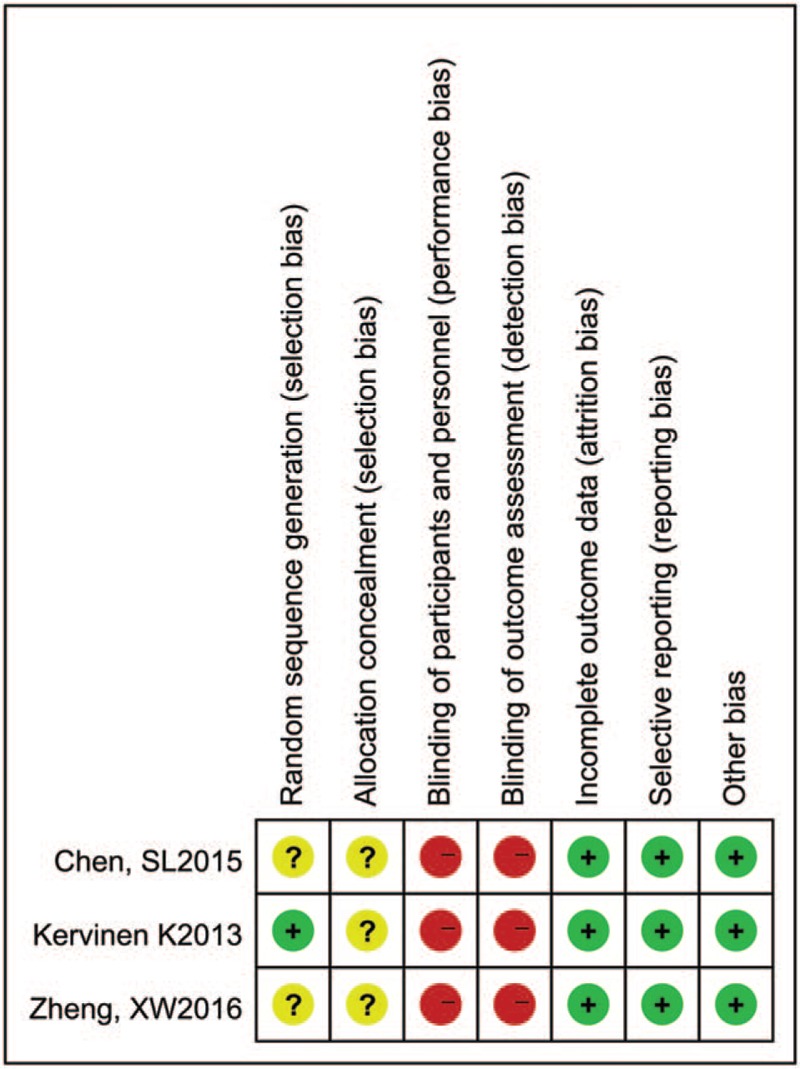
Risk of bias of the included randomized trials.

Patients characteristics were well matched between the 2 groups with weighted mean follow-up of 2.77 years, as shown in Table 1 and the clinical baseline characteristics in Table 2. The follow-up of endpoints was listed in Table 3. The methodological quality of the included studies was shown in Fig. 2 for randomized trials and in Table 4 for observational studies.

**Table 1:**
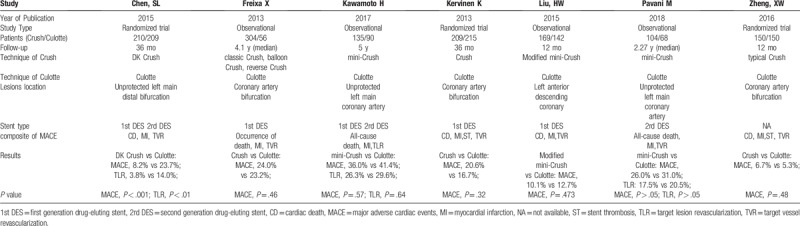
Characteristics of the 7 included studies.

**Table 2:**
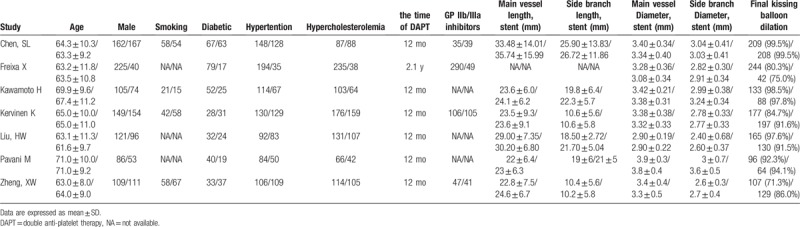
Clinical baseline characteristics of 7 included studies (Crush/Culotte).

**Table 3:**
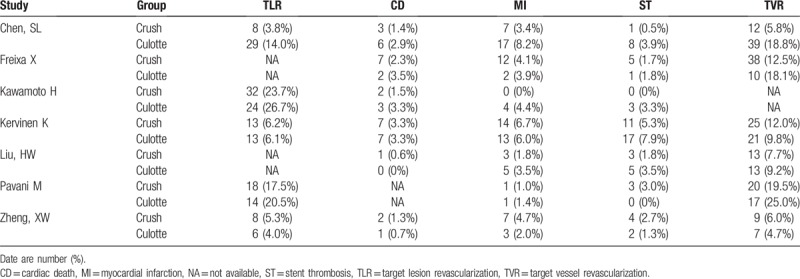
Follow-up of included 7 studies.

**Table 4:**
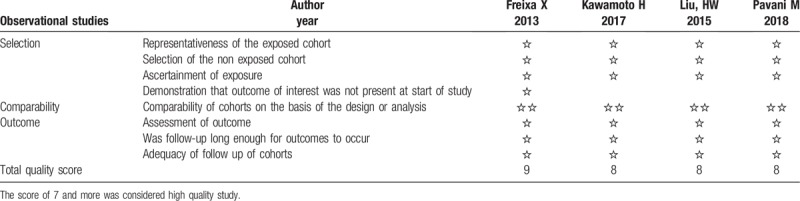
The Newcastle–Ottawa Scale for assessing the quality of observational studies.

### 3.2 Endpoints

MI and ST were assessed in all included studies. Owing to absence of the detail report of CD, we excluded the study by Pavani.^[8]^ Similarly, we excluded the study by Kawamoto,^[7]^ and the studies by Liu and Freixa,^[9,10]^ for TVR and TLR, respectively. In the same way, MACE were finally assessed in 5 included studies.^[4–6,9,10]^

**Figure 3:**
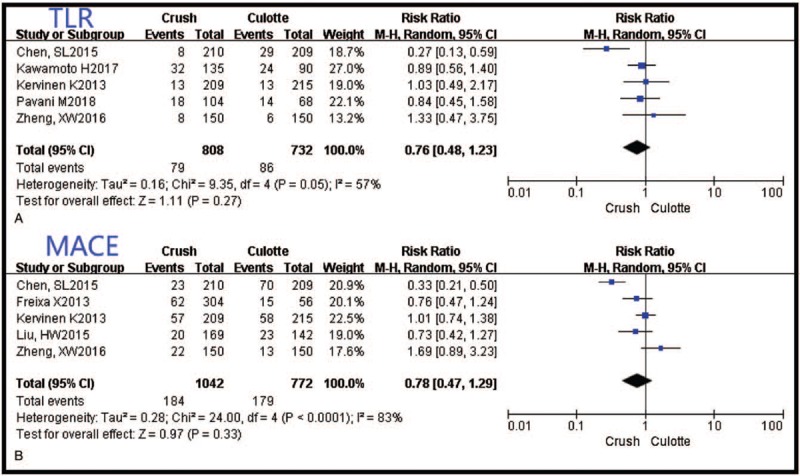
The forest plots of target lesion revascularization and major adverse cardiac events between the Crush and the Culotte groups. MACE = major adverse cardiac events, TLR = target lesion revascularization.

There was no significant difference in TLR and MACE between Crush and Culotte [RR 0.76, 95% CI (0.48–1.23), *P* = .27, I^2^ = 57%; RR 0.78, 95% CI (0.47–1.29), *P* = .33, I^2^ = 83%,respectively] (Fig. 3).

**Figure 4:**
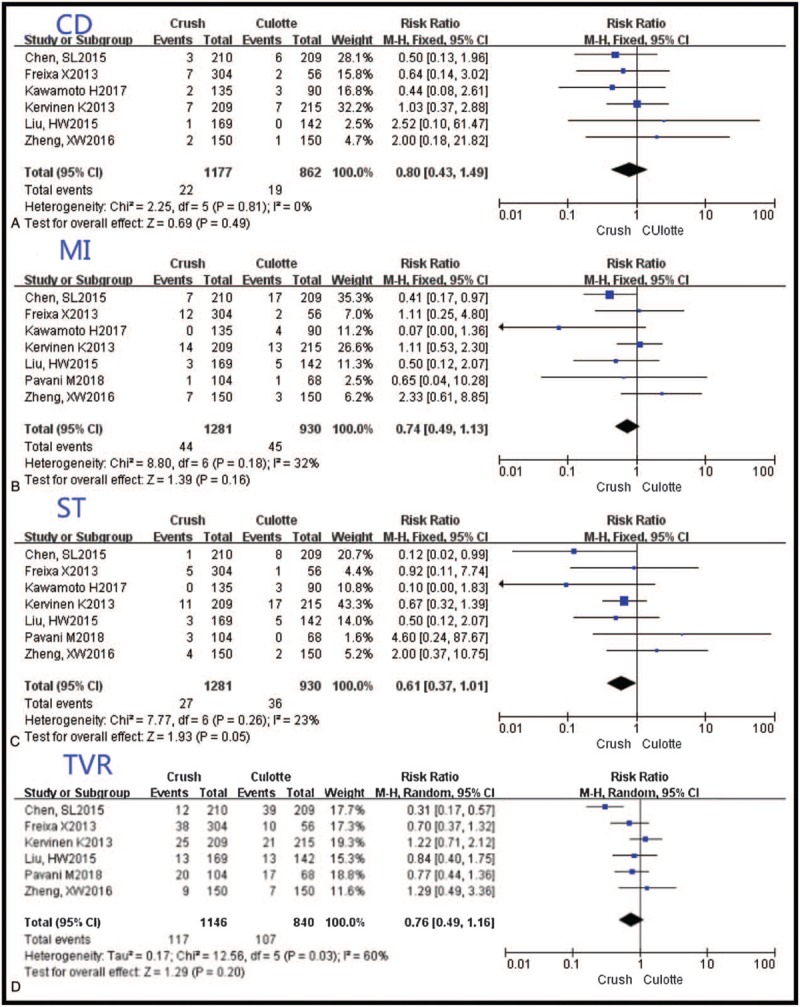
The forest plots of cardiac death, myocardial infarction, stent thrombosis, and target vessel revascularization between the Crush and the Culotte groups. CD = cardiac death, MI = myocardial infarction, ST = stent thrombosis, TVR = target vessel revascularization.

ST tended to be lower in patients treated with Crush [RR 0.61, 95% CI (0.37–1.01), *P* = .05, I^2^ = 23%]. CD and MI were comparable between the 2 groups [RR 0.80, 95% CI (0.43–1.49), *P* = .49, I^2^ = 0%; RR 0.74, 95% CI (0.49–1.13), *P* = .16, I^2^ = 32%, respectively]. And TVR was also associated with the similar risk [RR 0.76, 95% CI (0.49–1.16), *P* = .20, I^2^ = 60%] (Fig. 4).

### 3.3 Sensitivity analysis and publication bias

**Figure 5:**
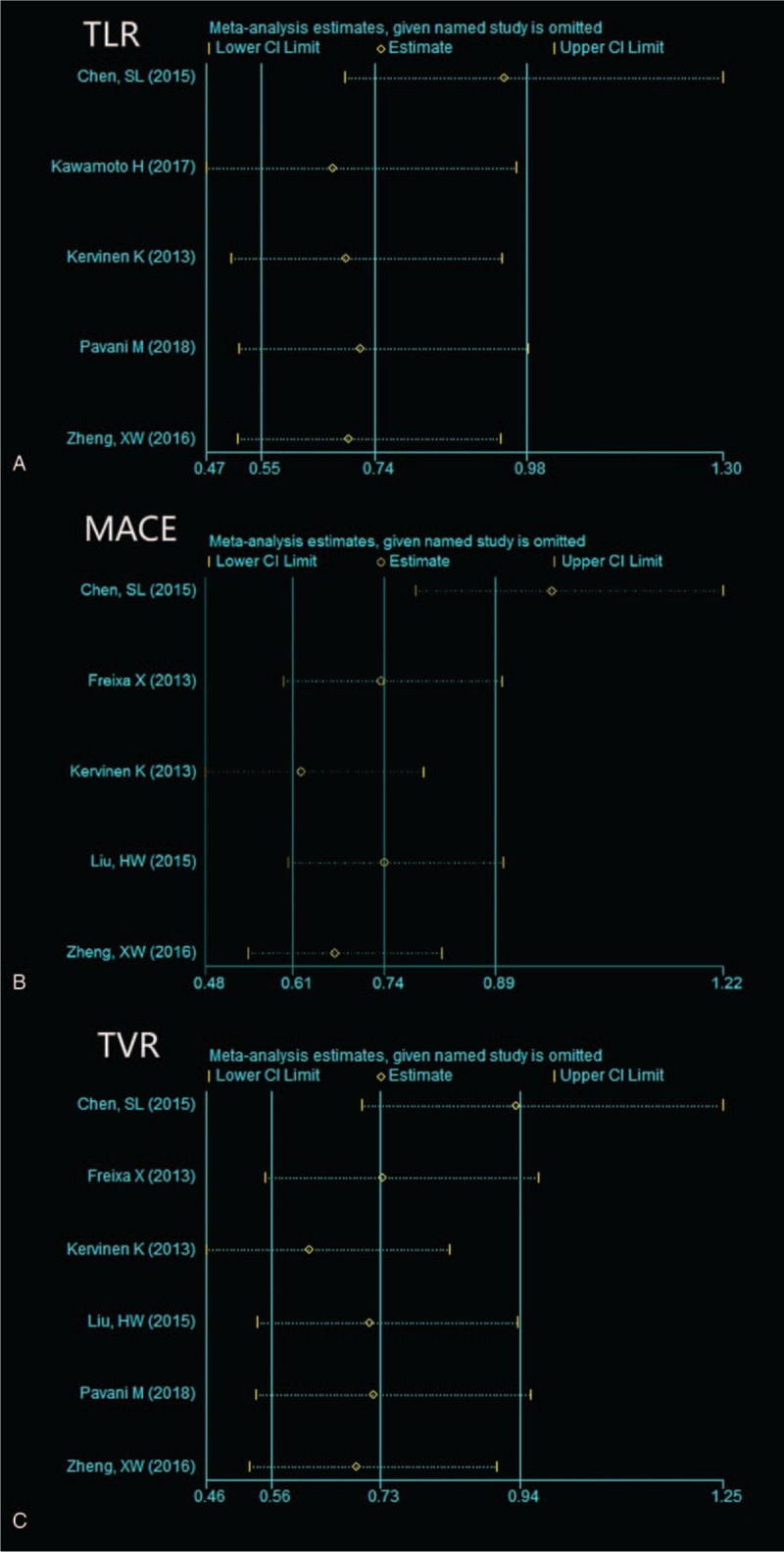
Sensitivity analysis of target lesion revascularization, major adverse cardiac events, and target vessel revascularization assessed by stata 14.0. MACE = major adverse cardiac events, TLR = target lesion revascularization, TVR = target vessel revascularization.

Since high heterogeneity was detected for TLR, MACE and TVR, we excluded each study in turn (Fig. 5, supplemental Table) and found that the source of heterogeneity was the study by Chen, SL.^[6]^ Publication bias (funnel plots shown in supplemental Figure 1) was not found in the included studies for TLR (Egger test t = −0.16, *P* = .885; Begg test z = 0.24, *P* = .806), and MACE (Egger test t = 0.06, *P* = .953; Begg test z = 0.24, *P* = 0.806).

There was also no publication bias in each individual component of MACE: CD (Egger test t = 0.68, *P* = .535; Begg test z = 0.75, *P* = .452), MI (Egger test t = −0.61, *P* = .570; Begg test z = 0.60, *P* = .548), ST (Egger test t = −0.14, *P* = .892; Begg test z = 0.00, *P* = 1.000), and TVR (Egger test t = 0.28, *P* = .793; Begg test z = 0.00, *P* = 1.000).

## 4 Discussion

This meta-analysis is the first assessing Crush and Culotte techniques for CBLs. Our main findings are: TLR, MACE, and TVR were comparable between Crush and Culotte techniques, but they were highly heterogeneous mainly due to better outcomes achieved by DK Crush than Culotte; CD and MI were similar between the 2 techniques; ST tended to be lower in Crush compared to Culotte. Overall, these results suggest that Crush, particularly DK Crush, may be superior to conventional Culotte for treatment of CBLs.

Since introducing of the stenting techniques of Crush by Colombo et al^[21]^ and Culotte by Chevalier et al^[22]^ for treatment of CBLs, both approaches have been optimized in several procedural steps, resulting in a family of Crush- or Culotte-based techniques. For Crush, the key modifications included prestaying a balloon (preferably bigger balloon) for crushing of SB stent before main-branch (MB) stent deployment (step-crush), shortening the crushed segment of the SB stent (mini-crush), and performing an intermediate balloon kissing dilation prior to MB stenting (DK-crush);^[23]^ while for Culotte, the major modifications consisted of stenting SB first (inverted culotte), prestaying a balloon in MB as stenting SB (MB balloon protection for procedural safety), shortening the overlapping segment of the 2 stents (mini-culotte), and performing an intermediate balloon kissing dilation prior to MB stenting (DK-culotte).^[24]^ Additionally, advent of the proximal optimization technique (POT) further optimizes the crush- or culotte-based techniques.^[25]^ All of which, as demonstrated by numerous studies, have significantly improved not only stenting techniques themselves but also their clinical outcomes.^[26–29]^ In this meta-analysis, the included studies were published from the year of 2013 to 2018, the Crush- or Culotte-based techniques used in this period experienced more or less technical optimization, probably leading to discrepancy in clinical outcomes as shown in this analysis.

TLR, associated closely with stenting technique itself, is generally accepted as a core index for the long-term efficacy of dual stenting techniques. In this meta-analysis, despite that TLR (the prespecified primary end-point), TVR and MACE were similar between Crush and Culotte, they were highly heterogeneous mainly due to the better outcomes achieved by DK Crush than Culotte as found in the sensitivity analysis. In DKCRUSH-III study, 3-year follow-up showed that compared to DK Crush, conventional Culotte was associated with increased MACE in patients with left main CBLs (23.7% vs 8.2%, *P* < .001), mostly driven by increased TLR (14.0% vs 3.8%, *P* < .001) or TVR (18.8% vs 5.8%, *P* < .001).^[6]^ DK Crush, as well known, is characterized by an intermediate kissing balloon dilation prior to MB stenting, which enable operators to more completely crush SB stent segment protruding into MB, remove the crushed struts over SB ostium and maintain fully expanding of the ostial SB stent, thereby facilitating subsequent wire or/and balloon crossing,^[30]^ and more importantly, final kissing balloon dilatation (FKBD).^[31,32]^ It has been confirmed that successful FKBD was associated with better long-term outcomes for all 2-stent techniques.^[5,6,33]^ A previous study reported that failed FKBD could lead to high occurrence of TLR in patients treated with Crush technique,^[34]^ while the Nordic Bifurcation Stent Technique Study showed that successful FKBD was associated with lower rates of MACE.^[5]^ As shown in the series of DK CRUSH trials and other observational studies, using DK-Crush could achieve more than 99% successful FKBD,^[6]^ which could well explain the better outcomes achieved by DK Crush than other Crush techniques or conventional Culotte.

ST, also associated closely with stenting technique itself, is usually considered another core index for the procedural safety of dual stenting techniques. Under the condition of no heterogeneity, this meta-analysis showed ST tended to be lower in Crush compared to Culotte irrespective of similar MI and CD between the 2 techniques. Again in DKCRUSH-III study, follow-up at 3 year revealed that compared with DK Crush, conventional Culotte was associated with increased definite ST (3.4% vs 0%, *P* = .007) and MI (8.2% vs 3.4%, *P* = .037).^[6]^ Also, the Nordic Bifurcation Stent Technique Study 36-month follow-up results showed that definite ST 1.4% in Crush and 4.7% in Culotte group (*P* = .09).^[5]^ These results together imply that Crush may be superior to Culotte in terms of the procedural safety. Technically, Crush distinguishes itself from Culotte in that the former has no and the latter has close interaction between 2-stents. For Crush stenting, SB stent is finally squeezed onto vascular wall by MB stent so that the relationship between 2-stents is only side-by-side contact;^[35]^ whereas for Culotte stenting, there is a tighter interaction between 2-stents (in the overlapped segment), probably causing stent–stent malposition (gaps between 2-stents) as used original Culotte, or stent-vessel malposition due to MB stent underexpansion restricted by side-hole of SB stent as used inverted Culotte. Such drawbacks occur frequently in case of significant diameter difference between branches or in use of limited expandability of stent platforms. Therefore, the different characteristics between Crush and Culotte may partially explain an increased risk of ST as using Culotte or inverted Culotte. Recently, by adding intermediate kissing balloon dilation prior to MB stenting when using inverted Culotte, DK mini-culotte and DK mono-ring culotte have been developed for treatment of true CBLs with promising clinical outcomes and potentially reducing the risk of ST in clinical studies.^[24,36,37]^

Obviously, there were some limitations in our study. First, we included only 3 randomized studies with relative small sample size. Nonetheless, the subgroup analysis according to study design (randomized vs observational) did not influence the results (supplemental Figure 2). Meanwhile, we also proved that there was not publication bias in the included studies and explained the potential source of heterogeneity. Second, we did not assess the effects of confounders on outcomes, such as CBLs characteristics (true CBLs, left main CBLs and high-angle CBLs), stent performance (first or second-generation of drug-eluting stents) and follow-up duration and so on. Third, we only roughly compared the Crush-based versus Culotte-based stenting techniques rather than parallel compared the corresponding techniques from these 2 families of dual stenting techniques (mini-Crush vs mini-Culotte, DK Crush vs DK Culotte). Due to these limitations, the conclusion that the Crush-based stenting is superior to Culotte-based stenting should be interpreted with cautions.

## 5 Conclusions

In the treatment of coronary bifurcation lesions, TLR and MACE were not significant difference between the Crush and Culotte groups, but TLR and MACE were also regarded as high heterogeneity mainly due to better outcomes achieved by DK Crush and there was a trend toward lower ST in the Crush group. Crush, particularly DK Crush, may be superior to conventional Culotte for treatment of CBLs. This possibly benefited from the technique of intermediate double balloon kissing dilation. When the technique introduces to the Culotte family (e.g., DK-culotte, DK-mini-culotte), the results perhaps be rewritten.

## Author contributions

EC: data collection and analysis, data interpretation, drafting manuscript, final critical revision of the manuscript, and final approval.

WC: data collection and analysis, data interpretation, drafting manuscript, final critical revision of the manuscript, and final approval.

L-LC: designer of the study, data analysis, data interpretation, drafting manuscript, final critical revision of the manuscript, and final approval.

**Conceptualization:** Liang-long Chen.

**Data curation:** En Chen, Wei Cai.

**Formal analysis:** Liang-long Chen, En Chen, Wei Cai.

**Funding acquisition:** Liang-long Chen.

**Investigation:** En Chen, Wei Cai.

**Methodology:** Liang-long Chen, En Chen, Wei Cai.

**Project administration:** En Chen, Wei Cai.

**Resources:** Wei Cai.

**Software:** En Chen, Wei Cai.

**Supervision:** Liang-long Chen.

**Writing – original draft:** En Chen, Wei Cai.

**Writing – review & editing:** Liang-long Chen.

Liang-long Chen orcid: 0000-0002-9281-6466.

## Supplementary Material

Supplemental Digital Content
