# Supplementary material for: Crush versus Culotte stenting techniques for coronary bifurcation lesions: A systematic review and meta-analysis of clinical trials with long-term follow-up
Source: Medicine (Baltimore). 2019 Apr 5;98(14):e14865. doi: 10.1097/MD.0000000000014865 (PMC6456007; doi:10.1097/MD.0000000000014865)
Supplement: Supplemental Digital Content [file medi-98-e14865-s001.docx]

**supplemental Table 1. Sensitivity analysis of target lesion revascularization, major adverse cardiac events and target vessel revascularization by omitting each study in turn**

| **TLR: sensitivity analysis** | | | |
| --- | --- | --- | --- |
| Removed Study | Year | I² | P value |
| Chen, SL | 2015 | 0 | 0.88 |
| Kawamoto H | 2017 | 66 | 0.03 |
| Kervinen K | 2013 | 66 | 0.03 |
| Pavani M | 2018 | 68 | 0.03 |
| Zheng, XW | 2016 | 64 | 0.04 |
| **MACE: sensitivity analysis** | | | |
| Removed Study | Year | I² | P value |
| Chen, SL | 2015 | 38 | 0.18 |
| Freixa X | 2013 | 88 | 0.0001 |
| Kervinen K | 2013 | 84 | 0.0003 |
| Liu, HW | 2015 | 87 | 0.0001 |
| Zheng, XW | 2016 | 83 | 0.0005 |
| **TVR: sensitivity analysis** | | | |
| Removed Study | Year | I² | P value |
| Chen, SL | 2015 | 0 | 0.61 |
| Freixa X | 2013 | 68 | 0.01 |
| Kervinen K | 2013 | 53 | 0.07 |
| Liu, HW | 2015 | 68 | 0.01 |
| Pavani M | 2018 | 68 | 0.01 |
| Zheng, XW | 2016 | 64 | 0.02 |

TLR: target lesion revascularization

MACE: major adverse cardiac events

TVR: target vessel revascularization


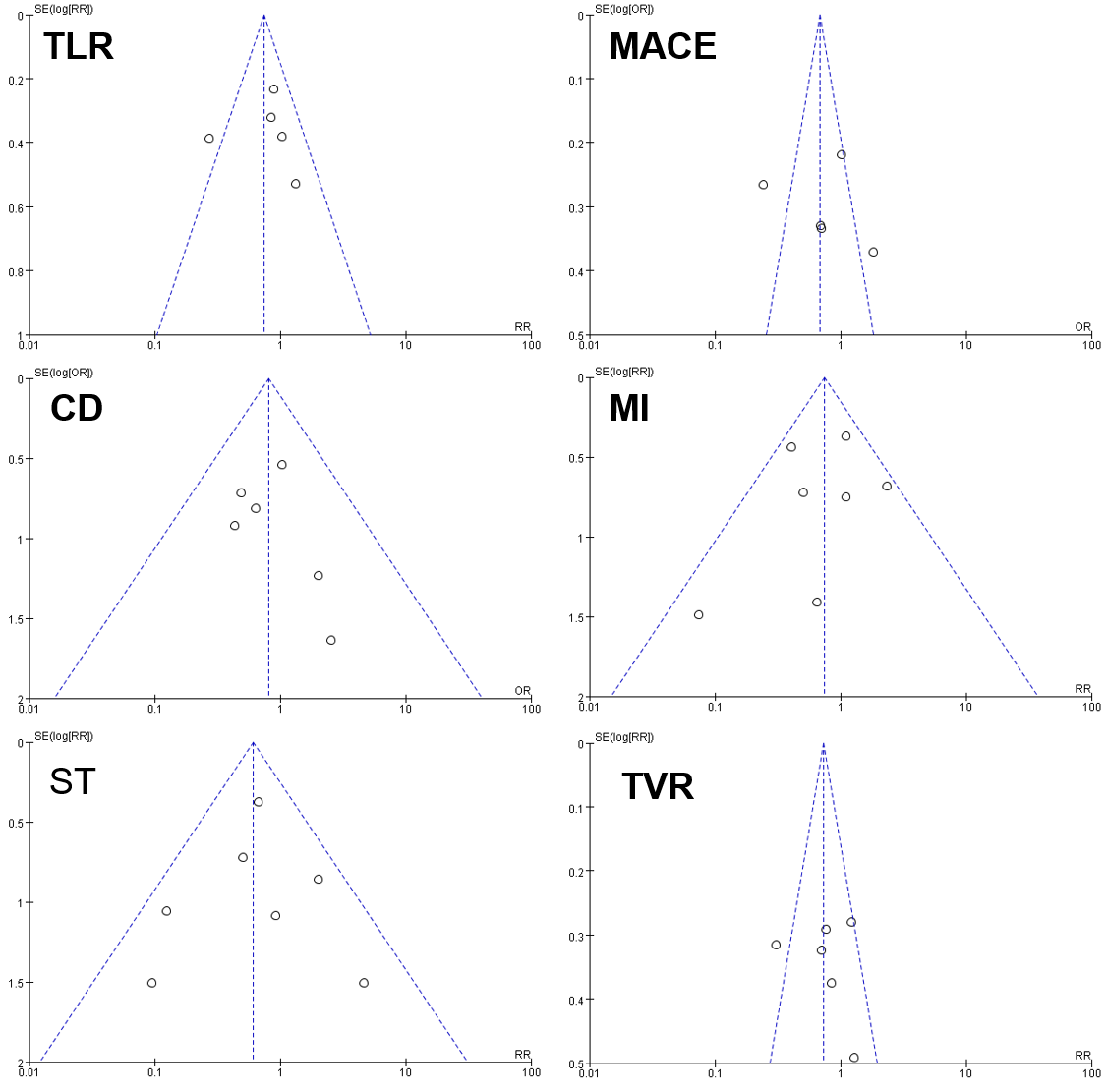


**Supplemental Figure 1. Funnel plots of TLR, MACE and each individual component of MACE**

TLR: target lesion revascularization; MACE: major adverse cardiac events; CD: cardiac death; MI: myocardial infarction; ST: stent thrombosis; TVR: target vessel revascularization.


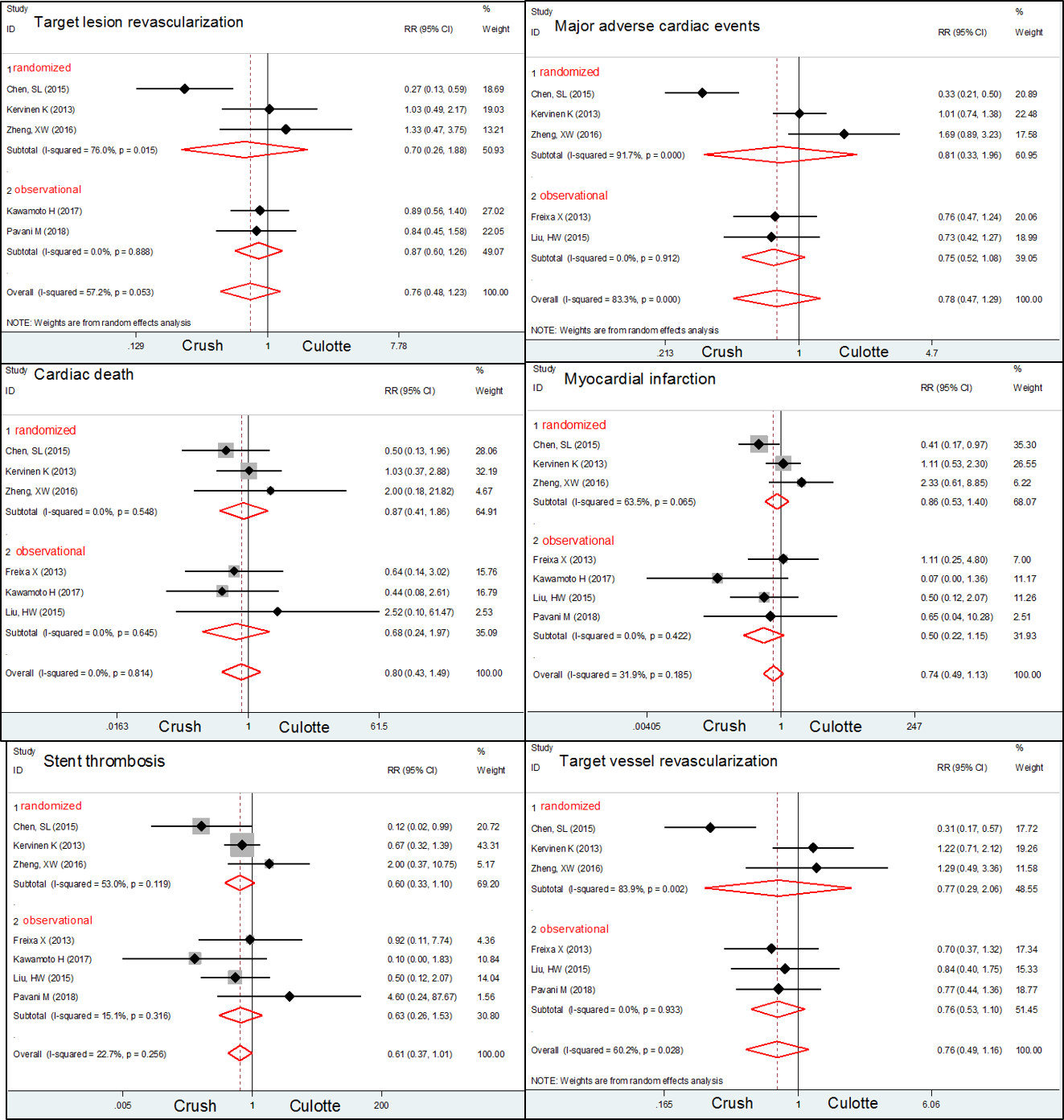


**Supplemental Figure 2.Subgroup analysis according to study design (randomized vs. observational)**

TLR: target lesion revascularization; MACE: major adverse cardiac events; CD: cardiac death; MI: myocardial infarction; ST: stent thrombosis; TVR: target vessel revascularization.
